# Genetic Markers of Adult Obesity Risk Are Associated with Greater Early Infancy Weight Gain and Growth

**DOI:** 10.1371/journal.pmed.1000284

**Published:** 2010-05-25

**Authors:** Cathy E. Elks, Ruth J. F. Loos, Stephen J. Sharp, Claudia Langenberg, Susan M. Ring, Nicholas J. Timpson, Andrew R. Ness, George Davey Smith, David B. Dunger, Nicholas J. Wareham, Ken K. Ong

**Affiliations:** 1 MRC Epidemiology Unit, Addenbrooke's Hospital, Cambridge, United Kingdom; 2 Institute of Metabolic Science, Addenbrooke's Hospital, Cambridge, United Kingdom; 3 Department of Social Medicine, University of Bristol, Bristol, United Kingdom; 4 MRC Centre for Causal Analyses in Translational Epidemiology, Department of Social Medicine, University of Bristol, Bristol, United Kingdom; 5 Department of Oral and Dental Science, University of Bristol, Bristol, United Kingdom; 6 Department of Paediatrics, University of Cambridge, Cambridge, United Kingdom; Children's Hospital Boston, United States of America

## Abstract

**Background:**

Genome-wide studies have identified several common genetic variants that are robustly associated with adult obesity risk. Exploration of these genotype associations in children may provide insights into the timing of weight changes leading to adult obesity.

**Methods and Findings:**

Children from the Avon Longitudinal Study of Parents and Children (ALSPAC) birth cohort were genotyped for ten genetic variants previously associated with adult BMI. Eight variants that showed individual associations with childhood BMI (in/near: *FTO, MC4R, TMEM18, GNPDA2, KCTD15, NEGR1, BDNF*, and *ETV5*) were used to derive an “obesity-risk-allele score” comprising the total number of risk alleles (range: 2–15 alleles) in each child with complete genotype data (*n* = 7,146). Repeated measurements of weight, length/height, and body mass index from birth to age 11 years were expressed as standard deviation scores (SDS). Early infancy was defined as birth to age 6 weeks, and early infancy failure to thrive was defined as weight gain between below the 5th centile, adjusted for birth weight. The obesity-risk-allele score showed little association with birth weight (regression coefficient: 0.01 SDS per allele; 95% CI 0.00–0.02), but had an apparently much larger positive effect on early infancy weight gain (0.119 SDS/allele/year; 0.023–0.216) than on subsequent childhood weight gain (0.004 SDS/allele/year; 0.004–0.005). The obesity-risk-allele score was also positively associated with early infancy length gain (0.158 SDS/allele/year; 0.032–0.284) and with reduced risk of early infancy failure to thrive (odds ratio  = 0.92 per allele; 0.86–0.98; *p* = 0.009).

**Conclusions:**

The use of robust genetic markers identified greater early infancy gains in weight and length as being on the pathway to adult obesity risk in a contemporary birth cohort.

*Please see later in the article for the Editors' Summary*

## Abstract 2

Ken Ong and colleagues genotyped children from the ALSPAC birth cohort and showed an association between greater early infancy gains in weight and length and genetic markers for adult obesity risk.

## Editors' Summary

**Background:**

The proportion of overweight and obese children is increasing across the globe. In the US, the Surgeon General estimates that, compared with 1980, twice as many children and three times the number of adolescents are now overweight. Worldwide, 22 million children under five years old are considered by the World Health Organization to be overweight.

Being overweight or obese in childhood is associated with poor physical and mental health. In addition, childhood obesity is considered a major risk factor for adult obesity, which is itself a major risk factor for cancer, heart disease, diabetes, osteoarthritis, and other chronic conditions.

The most commonly used measure of whether an adult is a healthy weight is body mass index (BMI), defined as weight in kilograms/(height in metres)^2^. However, adult categories of obese (>30) and overweight (>25) BMI are not directly applicable to children, whose BMI naturally varies as they grow. BMI can be used to screen children for being overweight and or obese but a diagnosis requires further information.

**Why Was This Study Done?:**

As the numbers of obese and overweight children increase, a corresponding rise in future numbers of overweight and obese adults is also expected. This in turn is expected to lead to an increasing incidence of poor health. As a result, there is great interest among health professionals in possible pathways between childhood and adult obesity. It has been proposed that certain periods in childhood may be critical for the development of obesity.

In the last few years, ten genetic variants have been found to be more common in overweight or obese adults. Eight of these have also been linked to childhood BMI and/or obesity. The authors wanted to identify the timing of childhood weight changes that may be associated with adult obesity. Knowledge of obesity risk genetic variants gave them an opportunity to do so now, without following a set of children to adulthood.

**What Did the Researchers Do and Find?:**

The authors analysed data gathered from a subset of 7,146 singleton white European children enrolled in the Avon Longitudinal Study of Parents and Children (ALSPAC) study, which is investigating associations between genetics, lifestyle, and health outcomes for a group of children in Bristol whose due date of birth fell between April 1991 and December 1992. They used knowledge of the children's genetic makeup to find associations between an obesity risk allele score—a measure of how many of the obesity risk genetic variants a child possessed—and the children's weight, height, BMI, levels of body fat (at nine years old), and rate of weight gain, up to age 11 years.

They found that, at birth, children with a higher obesity risk allele score were not any heavier, but in the immediate postnatal period they were less likely to be in the bottom 5% of the population for weight gain (adjusted for birthweight), often termed “failure to thrive.” At six weeks of age, children with a higher obesity risk allele score tended to be longer and heavier, even allowing for weight at birth.

After six weeks of age, the obesity risk allele score was not associated with any further increase in length/height, but it was associated with a more rapid weight gain between birth and age 11 years. BMI is derived from height and weight measurements, and the association between the obesity risk allele score and BMI was weak between birth and age three-and-a-half years, but after that age the association with BMI increased rapidly. By age nine, children with a higher obesity risk allele score tended to be heavier and taller, with more fat on their bodies.

**What Do These Findings Mean?:**

The combined obesity allele risk score is associated with higher rates of weight gain and adult obesity, and so the authors conclude that weight gain and growth even in the first few weeks after birth may be the beginning of a pathway of greater adult obesity risk.

A study that tracks a population over time can find associations but it cannot show cause and effect. In addition, only a relatively small proportion (1.7%) of the variation in BMI at nine years of age is explained by the obesity risk allele score.

The authors' method of finding associations between childhood events and adult outcomes via genetic markers of risk of disease as an adult has a significant advantage: the authors did not have to follow the children themselves to adulthood, so their findings are more likely to be relevant to current populations. Despite this, this research does not yield advice for parents how to reduce their children's obesity risk. It does suggest that “failure to thrive” in the first six weeks of life is not simply due to a lack of provision of food by the baby's caregiver but that genetic factors also contribute to early weight gain and growth.

The study looked at the combined obesity risk allele score and the authors did not attempt to identify which individual alleles have greater or weaker associations with weight gain and overweight or obesity. This would require further research based on far larger numbers of babies and children. The findings may also not be relevant to children in other types of setting because of the effects of different nutrition and lifestyles.

**Additional Information:**

Please access these Web sites via the online version of this summary at http://dx.doi.org/10.1371/journal.pmed.1000284.

- Further information is available on the ALSPAC study
- The UK National Health Service and other partners provide guidance on establishing a healthy lifestyle for children and families in their Change4Life programme
- The International Obesity Taskforce is a global network of expertise and the advocacy arm of the International Association for the Study of Obesity. It works with the World Health Organization, other NGOs, and stakeholders and provides information on overweight and obesity
- The Centers for Disease Control and Prevention (CDC) in the US provide guidance and tips on maintaining a healthy weight, including BMI calculators in both metric and Imperial measurements for both adults and children. They also provide BMI growth charts for boys and girls showing how healthy ranges vary for each sex at with age
- The Royal College of Paediatrics and Child Health provides growth charts for weight and length/height from birth to age 4 years that are based on WHO 2006 growth standards and have been adapted for use in the UK
- The CDC Web site provides information on overweight and obesity in adults and children, including definitions, causes, and data
- The CDC also provide information on the role of genes in causing obesity.
- The World Health Organization publishes a fact sheet on obesity, overweight and weight management, including links to childhood overweight and obesity
- Wikipedia includes an article on childhood obesity (note that Wikipedia is a free online encyclopedia that anyone can edit; available in several languages)

## Introduction

The increasing prevalence of overweight and obesity even in young preschool children [1] highlights the need to understand the very early determinants and potential targets for prevention of obesity. It has been proposed that there are certain critical periods in childhood for the development of obesity, including gestation and early infancy, the period of adiposity rebound between ages 5 and 7 years, and adolescence [2]. However, the relevance of overweight and obesity in infants and very young children to adult obesity and its comorbidities is unclear [3]. Common genetic variation associated with adult obesity may provide an opportunity to identify the timing of childhood weight changes that are associated with later obesity risk.

Recent technological advances and the massive increases in scale and statistical rigour of genome-wide association (GWA) studies has allowed the identification of common genetic variants associated with adult BMI and obesity risk that are consistently replicable in other populations. The first such common genetic variation shown to be associated with adult body mass index (BMI) was in the *FTO* gene region, published in 2007 by Frayling et al. [4]. This was closely followed by variation downstream of *MC4R* in 2008 [5]. Several further loci, in or near *TMEM18, GNPDA2, KCTD15, NEGR1, BDNF, ETV5, MTCH2*, and *SH2B1*, were recently reported to be adult obesity risk variants by studies from the GIANT international consortium [6] and the deCODE Genetics group [7].

To date all the published GWA-BMI studies primarily focussed on the association with adult BMI. The initial reports of common variants in/near to *FTO* and *MC4R* also included a demonstration of their relevance to childhood BMI and childhood obesity [4],[5], and these have since been confirmed in other childhood studies [8],[9]. A further four of the six new variants for adult BMI identified by the GIANT consortium also showed association with childhood BMI and/or childhood obesity (*TMEM18, GNPDA2, KCTD15*, and *NEGR1*) [6]. However, the joint publication by deCODE Genetics did not include any childhood populations [7].

Although cross-sectional associations with childhood BMI have been reported [4]–[6], the effects for most of these obesity risk variants on the rate of growth and weight gain during infancy and childhood have not yet been established. Data from the population-based Avon Longitudinal Study of Parents and Children (ALSPAC) birth cohort and other childhood obesity studies have shown the relevance of individual variants in *FTO* [4], *MC4R* [5], *TMEM18, GNPDA2, KCTD15*, and *NEGR1* [6] for childhood BMI and body fat mass. By further analysing these adult obesity susceptibility loci in the ALSPAC birth cohort, with the addition of variants in *BDNF* and *ETV5* [7], we aimed to identify the specific timing of childhood weight gain and growth associated with adult obesity risk. As the effects of individual variants are small (in the original studies of adult BMI the replication populations numbered in the tens of thousands [4]–[7]), we used a composite score of obesity risk alleles to maximise statistical power and reduce multiple testing.

## Methods

### Study Population

ALSPAC is a prospective study that has been described in detail elsewhere [10] (http://www.alspac.bris.ac.uk). Briefly, 14,541 pregnant women living in one of three Bristol-based health districts in the former County of Avon with an expected delivery date between April 1991 and December 1992 were enrolled in the study. Detailed information has been collected using self-administered questionnaires, data extraction from medical notes, and linkage to routine information systems and at research clinics. Ethical approval for the study was obtained from the ALSPAC Law and Ethics Committee and Local Research Ethics Committees.

### Growth Measurements

#### Infancy/early childhood

Birth weight as recorded in the delivery room was obtained from medical records. Birth length was measured by the study team. Infant and early childhood measures of weight and length (up to age 2 y) or height (from 2 y) at around ages 6 wk, 9 mo, and 1.5 and 3.5 y were available from routinely collected measurements performed by health visitors as part of the infant health surveillance programme and were extracted from the local child health database.

#### Later childhood

Childhood weight and height was measured annually between ages 7 and 11 y at dedicated ALSPAC Focus clinics by a trained research team. Height was measured to the nearest 0.1 cm using a Leicester Height Measure (Holtain Crosswell, Dyfed) and weight while wearing underwear was measured to the nearest 0.1 kg using Tanita electronic scales. Fat mass and fat-free mass was assessed at the 9-year-old research clinic visit by whole body dual energy X-ray absorptiometry (DXA) (Prodigy scanner, Lunar Radiation Corp, Madison, Wisconsin, US).

### Genotypes

Genotype information was available for six GWA-obesity variants previously reported to show association with BMI or obesity in children [4]–[6]; these variants were: rs9939609 (in/near to *FTO*); rs17782313 (*MC4R*), rs6548238 (*TMEM18*), rs10938397 (*GNPDA2*), rs368794 (*KCTD15*), rs2568958 (*NEGR1*). New genotype information was generated for two further variants reported to be associated with BMI in adults: rs925946 (*BDNF*) and rs7647305 (*ETV5*) [7]. Genotyping was performed by KBiosciences Ltd (Hoddesdon, UK) using their own novel system of fluorescence-based competitive allele-specific PCR (KASPar). Details of assay design are available from the KBiosciences Web site (http://www.kbioscience.co.uk). Call rates were 93.3% for rs925946 (*BDNF*) and 92.3% for rs7647305 (*ETV5*). All genotype frequencies met Hardy-Weinberg Equilibrium criteria (*p*>0.1).

### Calculations

BMI was calculated as weight (kg)/height^2^ (m^2^). Individual weight, length/height, and BMI values were converted to standard deviation scores (SDS) by comparison to the British 1990 growth reference, adjusted for sex and precise age at measurement [11],[12]. Values at birth were adjusted for gestational age using the growth reference. Children were classified as overweight or obese based on the International Obesity Task Force (IOTF) criteria, which uses population centile-based curves that pass through the 25 kg/m^2^ and 30 kg/m^2^ thresholds at age 18 y [13]. Fat and fat-free mass indices were calculated for each child from DXA measurements at age 9 y by dividing fat mass and fat-free mass (kg) by height squared (m^2^) [14].

Weight gain from birth to 6 wk, conditional on birth weight, was calculated using the following formula: 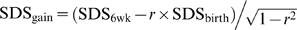 [15]. SDS_birth_ represents weight SDS at birth, SDS_6week_ weight SDS at the 6 wk measurement and *r* represents the population correlation between weight SDS at birth and 6 wk. The resulting SDS_gain_ represents a measure of weight gain from birth that is conditional on birth weight. Early infancy “failure to thrive” was defined as infants with the slowest 5% conditional weight gain from birth within the ALSPAC population [16].

### Statistical Analysis

Analyses were restricted to singleton white Europeans plus one randomly selected child from each mother for whom more than one child had entered the study. Linear regression was used to analyse whether common genetic variants in *BDNF* (rs925946) and *ETV5* (rs7647305) showed cross-sectional associations with BMI, weight, height, and body composition at 9 y.

An “obesity-risk-allele score” was created by counting the total number of obesity risk alleles across the eight variants that showed association with childhood weight or BMI (in/near to: *FTO, MC4R, TMEM18, GNPDA2, NEGR1, KCTD15, BDNF*, and *ETV5*). Only one variant at each locus was chosen and only individuals with complete genotype data at all eight variants were included in the obesity-risk-allele score analyses (*n* = 7,146 children). A “weighted obesity-risk-allele-score” (where contributions of each variant were weighted according to their apparent effect size on adult BMI) showed essentially the same associations as the currently reported unweighted score (unpublished data). For comparison, a second obesity-risk-allele score was created based on all ten variants (i.e., with the addition of the two variants in/near *SH2B1* and *MTCH2* with no evidence for association with childhood BMI or obesity [6]).

Linear regression was used to analyse the association between the obesity-risk-allele score and weight, height, and BMI SDS, and with fat mass index and fat-free mass index at age 9 y with adjustment for sex, precise age at measurement, and height. Logistic regression was used to analyse the association between the obesity-risk-allele score and the risks of infancy failure to thrive, and overweight or obesity at age 9 y.

Longitudinal analyses of associations between the obesity-risk-allele score and rates of weight gain were performed using the *xtmixed* command in STATA 10.1 to fit random intercepts models [17]. By assigning a unique identifier for each individual, this command performs a multi-level mixed effects linear regression analysis, allowing for clustering within individuals. A risk-score × age interaction term was calculated and added to the *xtmixed* models. This interaction term denotes by how much the effect of the obesity-risk-allele score gets stronger per year, and can be interpreted as the effect of the obesity-risk-allele score on linear change in weight SDS with age (in years). In further models, additional polynomial interaction terms (risk-score × age^2^ and risk-score × age^3^) were not significant (unpublished data). All analyses were conducted using STATA version 10.1 [17].

## Results

### The ALSPAC Population

Characteristics of the ALSPAC sample of children (*n* = 7,146) with complete genotype data are described in Table 1. Compared to other white European ALSPAC children without genotype data (*n* = 6,090), participants in this sample were slightly heavier at birth and showed modest differences in childhood BMI (<0.1 SDS difference at any age); their mothers were slightly older and more educated, but there was no difference in maternal BMI (see Table S1). The prevalence of obesity at age 9 y by IOTF criteria was 3.9% and 4.0% in boys and girls, respectively. At the same age, 20.0% of boys and 20.2% of girls met the IOTF criteria for overweight or obese (Table 1).

**Table 1:** Summary of growth measurements by age and sex.

*Variable*	Boys (*n* = 3610)			Girls (*n* = 3536)		
	*n*	*mean*	*(95% CI)*	*n*	*mean*	*(95% CI)*
**Weight (kg)**						
* Birth*	3441	3.49	(3.47–3.51)	3344	3.38	(3.37–3.40)
* 6 wk*	3124	5.22	(5.20–5.25)	3051	4.84	(4.81–4.86)
* 9 mo*	2839	9.53	(9.49–9.57)	2825	8.88	(8.84–8.92)
* 18 mo*	2658	12.2	(12.2–12.3)	2606	11.6	(11.6–11.7)
* 42 mo*	2712	16.7	(16.6–16.8)	2643	16.2	(16.1–16.3)
* 7 y*	2650	25.8	(25.6–25.9)	2608	25.8	(25.7–26.0)
* 8 y*	2193	30.1	(29.9–30.3)	2232	30.3	(30.0–30.5)
* 9 y*	2401	34.3	(34.0–34.6)	2478	34.9	(34.6–35.2)
* 10 y*	2314	37.6	(37.2–37.9)	2381	38.3	(38.0–38.6)
* 11 y*	2170	42.5	(42.1–42.9)	2294	44.5	(44.1–44.9)
**Length/Height (cm)**						
* Birth*	2737	51.1	(51.0–51.2)	2629	50.4	(50.3–50.5)
* 6 wk*	3003	58.0	(57.9–58.1)	2930	56.9	(56.8–57.0)
* 9 mo*	2881	73.2	(73.1–73.3)	2863	71.4	(71.3–71.5)
* 18 mo*	2723	84.5	(84.4–84.7)	2653	83.1	(83.0–83.2)
* 42 mo*	2708	100.9	(100.8–101.1)	2633	100.0	(99.8–100.1)
* 7 y*	2654	126.2	(126.0–126.4)	2611	125.5	(125.3–125.7)
* 8 y*	2277	132.9	(132.6–133.1)	2303	132.1	(131.8–132.3)
* 9 y*	2389	139.7	(139.5–140.0)	2452	139.4	(139.1–139.6)
* 10 y*	2307	143.9	(143.6–144.1)	2368	144.1	(143.9–144.4)
* 11 y*	2167	150.1	(149.8–150.4)	2295	151.5	(151.2–151.8)
**BMI (kg/m^2^)**						
* Birth*	2704	13.4	(7.1–25.6)	2601	13.3	(6.3–30.4)
* 6 wk*	2939	15.5	(15.4–15.5)	2860	14.9	(14.9–15.0)
* 9 mo*	2744	17.8	(17.7–17.8)	2736	17.4	(17.3–17.5)
* 18 mo*	2572	17.1	(17.0–17.1)	2512	16.8	(16.7–16.8)
* 42 mo*	2691	16.4	(16.3–16.4)	2624	16.2	(16.1–16.2)
* 7 y*	2650	16.1	(16.0–16.1)	2608	16.3	(16.2–16.40)
* 8 y*	2144	17.0	(16.9–17.0)	2180	17.3	(17.1–17.4)
* 9 y*	2387	17.5	(17.3–17.6)	2450	17.8	(17.7–18.0)
* 10 y*	2303	18.0	(17.9–18.1)	2363	18.3	(18.2–18.4)
* 11 y*	2293	18.7	(18.6–18.9)	2293	19.2	(19.1–19.4)
**Obese/Overweight** a		*Obese*	*Overweight/Obese*		*Obese*	*Overweight/Obese*
		3.9%	20.0%		4.0%	20.2%
**Fat mass index** a **(kg/m^2^)**	2285	3.7	(3.6–3.8)	2331	4.9	(4.8–4.9)
**Fat-free mass index** a **(kg/m^2^)**	2285	13.0	(13.0–13.1)	2331	12.1	(12.1–12.2)

a At age 9 y.

### Association of BDNF and ETV5 with Childhood BMI

Variants in rs925946 (*BDNF*) and rs7647305 (*ETV5*), which have not been previously studied in children, were associated with childhood BMI SDS, weight SDS, and height SDS (see Table S2). For example, at age 9 y each obesity-risk-allele at rs925946 was associated with +0.07 SDS (95% CI 0.03–0.12) greater childhood BMI, and +0.06 SDS (0.01–0.12) for rs7647305.

### The Obesity-Risk-Allele Score

**Figure 1:**
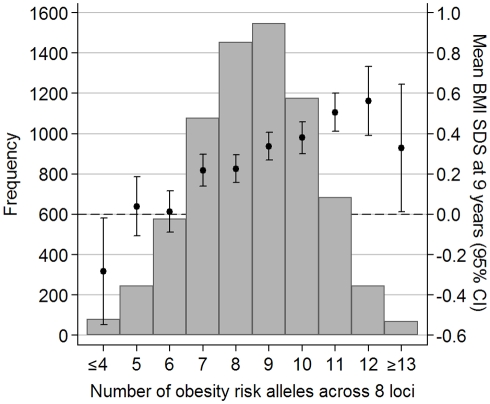
Distribution of the obesity-risk-allele score in ALSPAC children. The risk-allele score comprises genotypes at eight loci (*FTO, MC4R, TMEM18, GNPDA2, KCTD15, NEGR1, BDNF*, and *ETV5*). Error bars (and 2nd y-axis) display the mean (±95% CI) BMI SDS at age 9 y for each risk score value.

The obesity-risk-allele score based on genotypes at eight SNPs associated with childhood BMI (in/near to: *FTO, MC4R, TMEM18, GNPDA2, NEGR, KCTD15, BDNF*, and *ETV5*) ranged from 2 to 15 alleles. The score approximated a normal distribution and showed a linear association with BMI SDS at age 9 y (Figure 1). On average, each additional obesity risk allele conferred an estimated 0.07 SDS greater weight (95% CI 0.05–0.08; *p* = 1.2×10^−17^), 0.08 SDS greater BMI (0.06–0.10; *p* = 1.4×10^−19^), and 0.03 SDS greater height (0.01–0.04; *p* = 0.0003) at age 9 y. The differences in body size at age 9 y between the two extreme groups displayed in Figure 1 (≤4 risk alleles versus ≥13 risk-alleles) equated to 3.5 kg in body weight, 1.4 kg/m^2^ in BMI, and 2.0 cm in height at age 9 y. The obesity-risk-allele score explained 1.7% of the variance in BMI SDS at age 9 y (see Table S3). Furthermore, the obesity-risk-allele score was associated with increased risk of childhood overweight (odds ratio [OR] per allele  = 1.14; 95% CI 1.10–1.19; *p* = 6.3×10^−11^) and childhood obesity (OR = 1.17; 1.07–1.26; *p* = 0.0002) at age 9 years, and was more strongly associated with childhood fat mass index (0.13 kg/m^2^ per allele; 0.10–0.16, adjusted for sex, age, and height) than with fat-free mass index (0.03 kg/m^2^ per allele; 0.01–0.04) (Table 2).

**Table 2:** Association of the obesity-risk-allele score with measures of growth and adiposity at age 9 y.

Measure of Growth	*n*	Effect Size per Allele	95% CI	*P value*
BMI SDS	4837	0.08	(0.06–0.10)	*1.4×10^−19^*
Weight SDS	4879	0.07	(0.05–0.08)	*1.2×10^−17^*
Height SDS	4841	0.03	(0.01–0.04)	*2.5×10^−4^*
Fat mass index^a^ (kg/m^2^)	4616	0.13	(0.10–0.16)	*1.4×10^−13^*
Fat-free mass index^a^ (kg/m^2^)	4616	0.03	(0.01–0.04)	*2.9×10^−4^*

a *Adjusted for sex, age, and height.*

### Rate of Infancy and Childhood Weight Gain

**Figure 2:**
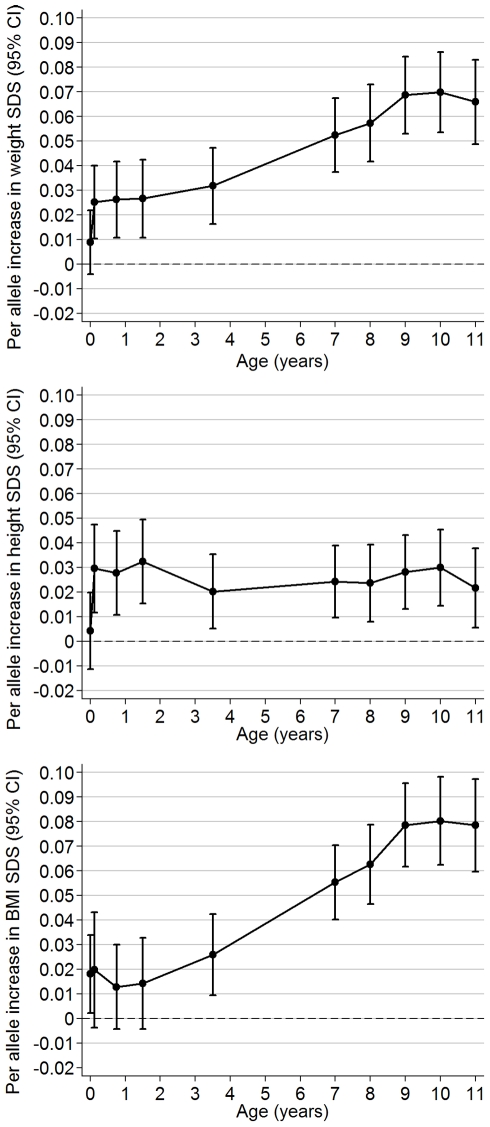
Longitudinal associations between the obesity-risk-allele score and (A) weight SDS, (B) length/height SDS, and (C) BMI SDS. Regression coefficients ±95% CI are shown from linear regression models (adjusted for sex and precise age at measurement) according to age at measurement between birth and 11 y.

The obesity-risk-allele score showed little association with birth weight SDS (effect size 0.01 SDS per allele; 95% CI 0.00–0.02; *p* = 0.2), but showed increasing positive associations with weight SDS from early infancy to childhood (Figure 2A). Even as early as age 6 wk, each additional risk allele was associated with a 0.03 SDS increase in weight (95% CI 0.01–0.04; *p* = 0.001).

Longitudinal analyses estimated that the obesity-risk-allele score was positively associated with rate of weight gain between birth to age 11 y (0.005 SDS/allele/y; 95% CI 0.004–0.006). The obesity-risk-allele score had an apparent much larger effect on the rate of early infancy weight gain (birth to 6 wk: 0.119 SDS/allele/y; 0.023–0.216) than on subsequent weight gain (6 wk to 11 y: 0.004 SDS/allele/y; 0.004–0.005).

The obesity-risk-allele score was positively associated with conditional weight gain between birth and age 6 wk (0.03 SDS gain per allele, 0.01–0.04; *p* = 0.001) and, conversely, was associated with reduced risk of early infancy “failure to thrive” between birth and age 6 wk (OR = 0.92 per allele, 0.86–0.98, *p* = 0.009).

### Childhood Length/Height and BMI

Similar to weight SDS, an association between the obesity-risk-allele score and length/height appeared soon after birth (association with length at 6 wk: 0.03 SDS/allele; 95% CI 0.01–0.05, *p* = 0.001). However, in contrast to weight, this association did not appear to increase in size subsequently during childhood (Figure 2B). Longitudinal analyses confirmed that the obesity-risk-allele score was positively associated with rate of gain in length during early infancy (birth to 6 wk: 0.158 SDS/allele/y; 0.032–0.284), but not subsequently (6 wk to 11 y: 0.000 SDS/allele/y; −0.001 to 0.001).

The obesity-risk-allele score was positively associated with BMI at birth (0.02 SDS/allele; 95% CI 0.00–0.03, *p* = 0.03), and longitudinal analyses showed a positive association with rate of gain in BMI between birth to age 11 y (0.006 SDS/allele/y; 0.005–0.007). However, the obesity-risk-allele score showed only weak association with BMI SDS during infancy until age 3.5 y onwards, from which time the association increased rapidly (Figure 2C).

### Comparison with the Ten-Variants Risk-Allele Score

Compared to the obesity-risk-allele score based on eight genetic variants, an extended obesity-risk-allele score based on ten variants (i.e., including genotypes at *SH2B1* and *MTCH2*, which were individually unrelated to childhood BMI) showed very similar, but slightly attenuated, associations with childhood body size and body composition (Table S4). The extended obesity-risk-allele score was also significantly associated with infant body weight at age 6 wk, and with conditional weight gain during the first 6 wk of life.

## Discussion

This study shows that recently established genetic variants for adult BMI have a combined association with childhood weight gain that is apparent even within the first weeks from birth. The combined association between these variants and childhood BMI, of around 0.5 SDS between the lowest and highest allele risk score groups, was similar in size to that seen with adult BMI (1.5 kg/m^2^) in terms of proportion of a standard deviation [6]. Therefore, while these risk variants may well influence rate of weight gain in adults [4], we postulate that that their relative influence on the rate of weight gain may be greater during childhood.

Their association with weight gain was already apparent from birth, within the first 6 weeks of life, and these adult obesity risk alleles were, in combination, protective against poor weight gain during the first weeks of life after birth. These findings are striking considering that these variants were originally discovered by association with adult BMI or obesity in populations with mean ages ranging from 40 to 60+ years [6],[7]. Other obesity susceptibility variants in/near *MAF*, *NPC1*, *PRL*, and *PTER* [18] have been identified in other GWA studies of early-onset and severe obesity, and putative associations with early life weight gain may be more expected with those variants. In addition, we observed that the adult obesity risk alleles were also associated with faster gains in length/height during infancy, but not during childhood. These findings are consistent with the Karlberg model of the endocrine regulation of childhood growth, whereby early infancy growth in length/height is largely controlled by nutritional factors, while the relatively stable trajectory of childhood growth reflects the setting of the growth hormone–insulin-like growth factor–I axis [19]. This potential weight-regulated drive in length gain likely explains the concurrent gains in both length and weight during infancy. Consequently, the obesity-risk-allele score showed a weaker association with BMI than with weight until age 3.5 years.

Rapid infancy weight gain and larger infant body weight have been consistently related to increased risk of obesity in subsequent childhood or adult life [20],[21]. However, life-course disease studies in historical cohort studies have provided conflicting evidence. For example, rapid infant weight gain has been associated with increased risk of obesity and the metabolic syndrome, but with reduced risk of type 2 diabetes [22]. One difficulty is that studies in historical cohorts or in societies that are undergoing nutritional transition may identify life-course associations that are specific to those particular settings [23]. Even in contemporary western studies, the very long-term follow-up needed to record adult disease outcomes may mean that current findings will not be applicable to future infant settings. We propose that the application in contemporary birth cohorts of genetic markers that are robustly associated with adult disease risks may provide a novel approach to life-course epidemiology, by identifying early exposures that are directly relevant to current settings.

Infancy weight gain and growth are markedly influenced by nutritional factors, such as type of milk feeding [24]. Our current findings show that genetic factors also contribute to early weight gain and growth. While the mechanisms of action for these obesity variants have yet to be established, many show high levels of expression, or have known actions, in the central nervous system and could therefore regulate feeding behaviour [6]. However, there is likely to be heterogeneity in their biological actions and in their specific effects during childhood and infancy. Future identification of the biological actions related to individual variants will shed further light on the specific early life mechanisms that lead to obesity. However, in view of their modest effect sizes, much larger studies or collaborations to pool longitudinal data from multiple birth cohorts will be needed to distinguish the specific childhood manifestations of individual variants.

Failure to thrive in infancy, variably defined as underweight or poor weight gain, is a multifactorial condition [25]. After exclusion of a wide range of medical conditions, nonorganic failure to thrive was traditionally considered to be a risk marker for maternal rejection and neglect [26]. However, the majority of infants with failure to thrive likely represent the lower-normal distribution of infancy weight gain. Health professionals have increasingly recognised the important contribution of innate differences in infant food intake rather than simply food provision by the caregiver [26], but until now no such infant factors have been demonstrated. We postulate that genetic factors in the infant that increase appetite and predispose to later obesity are protective against infant failure to thrive.

In conclusion, greater early infancy gains in weight and length represent the start of pathway to adult obesity risk in contemporary settings. Our findings demonstrate the utility of using robust genetic markers of disease risk to identify life-course disease associations with current relevance.

## Supporting Information

Table S1 Comparison of growth measurements in ALSPAC with complete genotype data (“Included”) and other white European ALSPAC children (“Excluded”).
(0.11 MB DOC)

Table S2 Association of variants in *BDNF* and *ETV5* with BMI, weight, and height SDS at each time point.
(0.11 MB DOC)

Table S3 Variance in weight SDS and BMI SDS explained by the obesity-risk-allele score at each time point.
(0.03 MB DOC)

Table S4 Comparison of obesity-risk-allele scores based on eight and ten genetic variants.
(0.06 MB DOC)

## Abbreviations

ALSPAC: Avon Longitudinal Study of Parents and Children
BMI: body mass index
DXA: dual energy X-ray absorptiometry
GWA: genome-wide association
OR: odds ratio
SDS: standard deviation scores
IOTF: International Obesity Task Force
