# Supplementary material for: Genetic Markers of Adult Obesity Risk Are Associated with Greater Early Infancy Weight Gain and Growth
Source: PLoS Med. 2010 May 25;7(5):e1000284. doi: 10.1371/journal.pmed.1000284 (PMC2876048; doi:10.1371/journal.pmed.1000284)
Supplement: Table S1 — Comparison of growth measurements in ALSPAC with complete genotype data (“Included”) and other white European ALSPAC children (“Excluded”). (0.11 MB DOC) [file pmed.1000284.s001.doc]

**Supplementary Table 1: Comparison of growth measurements in ALSPAC with complete genotype data (“Included”) and other white European ALSPAC children (“Excluded”).**

|  | **Included (n=7146)** | | | **Excluded (n=6090)** | | | *p value* |
| --- | --- | --- | --- | --- | --- | --- | --- |
|  | *n* | *mean* | *(95% CI)* | *n* | *mean* | *(95% CI)* |  |
| *Weight SDS* |  |  |  |  |  |  |  |
| Birth | 6785 | 0.14 | (0.12-0.16) | 5843 | 0.06 | (0.04-0.09) | ***<0.001*** |
| 6 weeks | 6175 | 0.15 | (0.12-0.17) | 5161 | 0.11 | (0.09-0.14) | *0.13* |
| 9 months | 5664 | 0.32 | (0.29-0.34) | 4667 | 0.30 | (0.27-0.33) | *0.56* |
| 18 months | 5264 | 0.45 | (0.42-0.47) | 4295 | 0.43 | (0.40-0.47) | *0.63* |
| 42 months | 5351 | 0.28 | (0.24-0.29) | 4318 | 0.25 | (0.22-0.29) | *0.55* |
| *7y* | 5258 | 0.22 | (0.19-0.25) | 2400 | 0.26 | (0.22-0.30) | *0.13* |
| *8y* | 4425 | 0.39 | (0.37-0.42) | 2084 | 0.41 | (0.36-0.45) | *0.65* |
| *9y* | 4879 | 0.36 | (0.33-0.39) | 2314 | 0.39 | (0.35-0.43) | *0.29* |
| *10y* | 4695 | 0.40 | (0.37-0.43) | 2327 | 0.42 | (0.38-0.47) | *0.37* |
| *11y* | 4464 | 0.51 | (0.48-0.54) | 2192 | 0.55 | (0.51-0.60) | *0.13* |
|  |  |  |  |  |  |  |  |
| *Height/length SDS* |  |  |  |  |  |  |  |
| Birth | 5332 | 0.25 | (0.22-0.28) | 4299 | 0.22 | (0.19-0.26) | *0.24* |
| 6 weeks | 5933 | 0.53 | (0.39-0.45) | 4926 | 0.49 | (0.46-0.53) | *0.13* |
| 9 months | 5744 | 0.54 | (0.51-0.57) | 4772 | 0.52 | (0.49-0.56) | *0.4* |
| 18 months | 5376 | 0.54 | (0.51-0.57) | 4356 | 0.50 | (0.47-0.54) | *0.12* |
| 42 months | 5336 | 0.06 | (0.03-0.09) | 4301 | 0.004 | (-0.03-0.03) | ***0.008*** |
| *7y* | 5265 | 0.24 | (0.19-0.26) | 2406 | 0.21 | (0.17-0.25) | *0.23* |
| *8y* | 4580 | 0.27 | (0.25-0.30) | 2143 | 0.24 | (0.20-0.28) | *0.2* |
| *9y* | 4841 | 0.32 | (0.29-0.25) | 2296 | 0.3 | (0.26-0.34) | *0.46* |
| *10y* | 4675 | 0.35 | (0.33-0.38) | 2321 | 0.31 | (0.27-0.35) | *0.12* |
| *11y* | 4462 | 0.47 | (0.44-0.50) | 2190 | 0.43 | (0.39-0.47) | *0.15* |
|  |  |  |  |  |  |  |  |
| *BMI SDS* |  |  |  |  |  |  |  |
| *7y* | 5258 | 0.12 | (0.09-0.15) | 2399 | 0.20 | (0.16-0.25) | ***0.001*** |
| *8y* | 4324 | 0.37 | (0.34-0.40) | 2027 | 0.41 | (0.36-0.45) | *0.15* |
| *9y* | 4837 | 0.28 | (0.25-0.32) | 2295 | 0.34 | (0.29-0.38) | *0.06* |
| *10y* | 4666 | 0.30 | (0.27-0.33) | 2313 | 0.36 | (0.32-0.41) | ***0.03*** |
| *11y* | 4460 | 0.34 | (0.30-0.37) | 2188 | 0.42 | (0.37-0.47) | ***0.008*** |
|  |  |  |  |  |  |  |  |
| *Mother’s BMI* | 6015 | 23.0 | (22.9-23.0) | 4747 | 23.0 | (22.9-23.0) | *0.85* |
| *Mother’s age at delivery* | 6868 | 28.5 | (28.4-28.6) | 5927 | 27.5 | (27.4-27.6) | ***<0.001*** |
| *Mother’s highest education* |  |  |  |  |  |  |  |
| *- CSE* | 1091 | 16.9% |  | 1217 | 23.5% |  |  |
| *- Vocational* | 598 | 9.3% |  | 545 | 10.5% |  |  |
| *- O level* | 2282 | 35.3% |  | 1803 | 34.8% |  |  |
| *- A level* | 1546 | 23.9% |  | 1071 | 20.6% |  |  |
| *- Degree* | 940 | 14.6% |  | 542 | 10.4% |  | ***<0.001*** |
