# Supplementary material for: Genetic Markers of Adult Obesity Risk Are Associated with Greater Early Infancy Weight Gain and Growth
Source: PLoS Med. 2010 May 25;7(5):e1000284. doi: 10.1371/journal.pmed.1000284 (PMC2876048; doi:10.1371/journal.pmed.1000284)
Supplement: Table S2 — Association of variants in BDNF and ETV5 with BMI, weight, and height SDS at each time point. (0.11 MB DOC) [file pmed.1000284.s002.doc]

**Supplementary Table 2: Association of variants in *BDNF* and *ETV5* with BMI, weight and height SDS at each timepoint.**

| Nearby gene | SNP | Chr | Alleles | | Frequency of effect allele | | Age | | BMI SDS | | | | | | | Weight SDS | | | | | | | Height SDS | | | | | |
| --- | --- | --- | --- | --- | --- | --- | --- | --- | --- | --- | --- | --- | --- | --- | --- | --- | --- | --- | --- | --- | --- | --- | --- | --- | --- | --- | --- | --- |
|  |  |  | *Effect* | *Other* |  |  |  |  | *beta* | | *95% CI* | | *P value* | | *beta* | | | *95% CI* | | *P value* | | *beta* | | | *95% CI* | | *P value* | |
| *BDNF* | rs925946 | 11 | T | G | 31% | *Birth* | | 0.01 | | -0.04-0.05 | | *0.71* | | 0.00 | | | -0.03-0.04 | | *0.79* | | 0.02 | | | -0.02-0.08 | | *0.31* | |  |
|  |  |  |  |  |  | *6wk* | | 0.04 | | -0.02-0.10 | | *0.20* | | 0.04 | | | 0.00-0.08 | | ***0.04*** | | 0.03 | | | -0.02-0.08 | | *0.25* | |  |
|  |  |  |  |  |  | *9mo* | | 0.02 | | -0.03-0.06 | | *0.48* | | 0.04 | | | *-0.00-0.082* | | *0.05* | | 0.03 | | | -0.01-0.80 | | *0.15* | |  |
|  |  |  |  |  |  | *18mo* | | 0.08 | | 0.03-0.13 | | ***0.003*** | | 0.06 | | | 0.02-0.11 | | ***0.003*** | | 0.02 | | | -0.03-0.07 | | *0.41* | |  |
|  |  |  |  |  |  | *42mo* | | 0.05 | | 0.00-0.09 | | ***0.04*** | | 0.06 | | | 0.02-0.10 | | ***0.008*** | | 0.04 | | | 0.00-0.09 | | ***0.03*** | |  |
|  |  |  |  |  |  | *7y* | | 0.06 | | 0.02-0.10 | | ***0.002*** | | 0.05 | | | 0.01-0.20 | | ***0.004*** | | 0.03 | | | -0.01-0.07 | | *0.12* | |  |
|  |  |  |  |  |  | *8y* | | 0.05 | | 0.01-0.09 | | ***0.008*** | | 0.06 | | | 0.02-0.10 | | ***0.004*** | | 0.05 | | | 0.00-0.09 | | ***0.03*** | |  |
|  |  |  |  |  |  | *9y* | | 0.07 | | 0.03-0.12 | | ***0.001*** | | 0.08 | | | 0.04-0.12 | | ***0.0003*** | | 0.05 | | | 0.01-0.09 | | ***0.02*** | |  |
|  |  |  |  |  |  | *10y* | | 0.07 | | 0.03-0.11 | | ***0.002*** | | 0.07 | | | 0.03-0.12 | | ***0.001*** | | 0.05 | | | 0.01-0.09 | | ***0.02*** | |  |
|  |  |  |  |  |  | *11y* | | 0.06 | | 0.02-0.11 | | ***0.007*** | | 0.08 | | | 0.03-0.12 | | ***0.001*** | | 0.05 | | | 0.01-0.09 | | ***0.03*** | |  |
| *ETV5* | rs7647305 | 3 | C | T | 80% | *Birth* | | 0.02 | | -0.03-0.07 | | *0.44* | | 0.01 | | | -0.03-0.06 | | *0.47* | | 0.00 | | | -0.05-0.05 | | *0.97* | |  |
|  |  |  |  |  |  | *6wk* | | 0.08 | | 0.01-0.16 | | ***0.02*** | | 0.04 | | | -0.01-0.09 | | *0.10* | | 0.007 | | | -0.05-0.06 | | *0.79* | |  |
|  |  |  |  |  |  | *9mo* | | 0.04 | | -0.01-0.09 | | *0.16* | | 0.04 | | | -0.01-0.09 | | *0.11* | | 0.03 | | | -0.03-0.08 | | *0.35* | |  |
|  |  |  |  |  |  | *18mo* | | 0.05 | | -0.01-0.11 | | *0.11* | | 0.06 | | | 0.01-0.11 | | ***0.02*** | | 0.03 | | | -0.02-0.09 | | *0.24* | |  |
|  |  |  |  |  |  | *42mo* | | 0.04 | | -0.01-0.10 | | *0.10* | | 0.06 | | | 0.02-0.11 | | ***0.01*** | | 0.05 | | | -0.00-0.09 | | *0.06* | |  |
|  |  |  |  |  |  | *7y* | | 0.06 | | 0.02-0.11 | | ***0.008*** | | 0.08 | | | 0.03-0.12 | | ***0.002*** | | 0.06 | | | 0.01-0.10 | | ***0.02*** | |  |
|  |  |  |  |  |  | *8y* | | 0.06 | | 0.01-0.11 | | ***0.03*** | | 0.06 | | | 0.01-0.11 | | ***0.01*** | | 0.04 | | | -0.01-0.09 | | *0.14* | |  |
|  |  |  |  |  |  | *9y* | | 0.07 | | 0.01-0.12 | | ***0.01*** | | 0.08 | | | 0.03-0.12 | | ***0.003*** | | 0.06 | | | 0.01-0.11 | | ***0.01*** | |  |
|  |  |  |  |  |  | *10y* | | 0.07 | | 0.02-0.13 | | ***0.01*** | | 0.07 | | | 0.02-0.13 | | ***0.006*** | | 0.05 | | | -0.00-0.09 | | *0.07* | |  |
|  |  |  |  |  |  | *11y* | | 0.08 | | 0.02-0.15 | | ***0.006*** | | 0.07 | | | 0.02-0.13 | | ***0.009*** | | 0.03 | | | -0.02-0.09 | | *0.20* | |  |
