# Supplementary material for: Genetic Markers of Adult Obesity Risk Are Associated with Greater Early Infancy Weight Gain and Growth
Source: PLoS Med. 2010 May 25;7(5):e1000284. doi: 10.1371/journal.pmed.1000284 (PMC2876048; doi:10.1371/journal.pmed.1000284)
Supplement: Table S3 — Variance in weight SDS and BMI SDS explained by the obesity-risk-allele score at each time point. (0.03 MB DOC) [file pmed.1000284.s003.doc]

**Supplementary Table 3: Variance in weight SDS and BMI SDS explained by the obesity risk-allele score at each timepoint**

| **Age** | **Weight SDS variance explained (%)** | **BMI SDS**  **variance explained (%)** |
| --- | --- | --- |
| Birth | 0.0 | 0.1 |
| 6 weeks | 0.2 | 0.1 |
| 9 months | 0.2 | 0.0 |
| 18 months | 0.2 | 0.0 |
| 42 months | 0.2 | 0.2 |
| 7y | 0.9 | 1.0 |
| 8y | 1.1 | 1.3 |
| 9y | 1.5 | 1.7 |
| 10y | 1.5 | 1.6 |
| 11y | 1.2 | 1.5 |
