# Supplementary material for: Genetic Markers of Adult Obesity Risk Are Associated with Greater Early Infancy Weight Gain and Growth
Source: PLoS Med. 2010 May 25;7(5):e1000284. doi: 10.1371/journal.pmed.1000284 (PMC2876048; doi:10.1371/journal.pmed.1000284)
Supplement: Table S4 — Comparison of obesity-risk-allele scores based on eight and ten genetic variants. (0.06 MB DOC) [file pmed.1000284.s004.doc]

**Supplementary Table 4: Comparison of obesity-risk-allele scores based on 8 and 10 genetic variants**

**Associations with body size and body composition at age 9 years**

|  | ***8-variant risk allele score^1^*** | | | | | | | ***10-variant risk allele score^2^*** | | | | |
| --- | --- | --- | --- | --- | --- | --- | --- | --- | --- | --- | --- | --- |
|  | n | Effect per allele | 95% CI | | *P value* | | | n | | Effect per allele | 95% CI |  |
|  |  |  |  |  |  |  |  |  |  |  |  | *P value* |
| BMI SDS | 4837 | 0.08 | (0.06-0.10) | | *1.4x10^-19^* | | | 4722 | | 0.07 | (0.05-0.08) | *2.7x10^-18^* |
| Weight SDS | 4879 | 0.07 | (0.05-0.08) | | *1.2x10^-17^* | | | 4763 | | 0.05 | (0.04-0.07) | *1.8x10^-14^* |
| Height SDS | 4841 | 0.03 | (0.01-0.04) | | *2.5x10^-4^* | | | 4726 | | 0.02 | (0.00-0.03) | *0.01* |
| Fat mass index* (kg/m^2^) | 4616 | 0.13 | (0.10-0.16) | | *1.4x10^-13^* | | | 4505 | | 0.12 | (0.09-0.15) | *3.0x10^-14^* |
| Fat-free mass index* (kg/m^2^) | 4616 | 0.03 | (0.01-0.04) | | *2.9x10^-4^* | | | 4505 | | 0.02 | (0.01-0.03) | *0.001* |
| Overweight (odds ratio) | 4837 | 1.14 | (1.10-1.19) | | *6.3x10^-11^* | | | 4722 | | 1.12 | (1.08-1.16) | *2.0x10^-10^* |
| Obesity (odds ratio) | 4837 | 1.17 | (1.07-1.26) | | *2.0x10^-4^* | | | 4722 | | 1.17 | (1.09-1.26) | *2.0x10^-5^* |
| **adjusted for sex, age and height* | |  | |  |  |  |  | |  | | | |

**Associations with birth weight, infant weight, conditional infant weight gain and failure to thrive (between birth to age 6 weeks)**

|  | ***8-variant risk allele score^1^*** | | | | ***10-variant risk allele score^2^*** | | | |
| --- | --- | --- | --- | --- | --- | --- | --- | --- |
|  | n | Effect per allele | 95% CI | *P value* | n | Effect per allele | 95% CI |  |
|  |  |  |  |  |  |  |  | *P value* |
| Birth weight (SDS) | 6785 | 0.01 | (-0.00-0.02) | 0.2 | 6597 | 0.00 | (-0.01-0.01) | 0.8 |
| Weight at age 6 weeks (SDS) | 6175 | 0.03 | 0.01-0.04 | 0.001 | 6005 | 0.02 | (0.00-0.03) | 0.02 |
| Conditional weight gain (SDS) | 6070 | 0.03 | (0.01-0.04) | *0.001* | 5903 | 0.02 | (0.01-0.03) | *0.004* |
| Failure to thrive  (odds ratio) | 6070 | 0.92 | (0.86-0.98) | *0.009* | 5903 | 0.94 | (0.89-1.00) | *0.038* |

^1^Comprising genotypes in SNPs in/near: *FTO, MC4R, TMEM18, GNPDA2, KCTD15, NEGR1, BDNF* and *ETV5*

^2^As above with the addition of SNPs in/near: *SH2B1* and *MTCH2*
